# Combination of Brentuximab Vedotin and Pembrolizumab as Salvage Treatment Before Autologous Stem Cell Transplantation and Maintenance in Patients with Relapsed/Refractory Hodgkin Lymphoma †

**DOI:** 10.3390/biomedicines13020252

**Published:** 2025-01-21

**Authors:** Hanne Massa, Fulvio Massaro, Marie Maerevoet

**Affiliations:** Department of Clinical Hematology, Institut Jules Bordet (HUB), 1070 Brussels, Belgium; fulvio.massaro@hubruxelles.be (F.M.); marie.maerevoet@hubruxelles.be (M.M.)

**Keywords:** Hodgkin lymphoma, antibody–drug conjugate, autologous stem cell transplantation, brentuximab vedotin, immune checkpoint inhibition, pembrolizumab, salvage therapy, relapsed/refractory

## Abstract

**Background:** In relapsed or refractory classical Hodgkin lymphoma, achieving complete remission on 18FDG PET-CT before autologous stem cell transplantation improves progression-free survival. However, the optimal salvage therapy to achieve this remains undefined. Brentuximab vedotin combined with PD1 inhibitors has shown promise, though limited data exist on the combination of brentuximab vedotin and pembrolizumab. **Methods:** We retrospectively collected data from 24 adult patients with confirmed relapsed or refractory classical Hodgkin lymphoma, who started salvage treatment with brentuximab vedotin and pembrolizumab with the intention of consolidation with high-dose chemotherapy, followed by autologous stem cell transplantation and brentuximab vedotin maintenance. **Results:** After two cycles of brentuximab vedotin and pembrolizumab, 95.2% achieved an overall response and 81.0% achieved complete metabolic response. 20 patients (83.3%) were in complete response at the end of maintenance, of whom one relapsed at 28 months after the end of treatment. Grade 3 and 4 toxicities during salvage treatment consisted mainly of hematological toxicity, one thyrotoxicosis, one hemophagocytic lymphohistiocytosis, and one arthralgia. Non-hematological grade 3–4 toxicities following transplantation were an inflammatory pneumonitis and one cryptococcal meningitis. One death occurred during prolonged post-transplant aplasia. During maintenance, dose reductions for toxicity were necessary in 16 patients, mainly due to peripheral neuropathy. **Conclusions:** For heavily pretreated relapsed or refractory classical Hodgkin lymphoma patients, our data suggest that salvage therapy with brentuximab vedotin and pembrolizumab before autologous stem cell transplantation followed by brentuximab vedotin maintenance is a highly active strategy, with acceptable toxicities. Further studies with larger cohorts are necessary to confirm these data.

## 1. Introduction

Even though classical Hodgkin lymphoma (cHL) is a highly curable disease, 2–30% of patients suffer from relapse or refractory disease after first-line treatment [1]. The current standard for the treatment of relapsed or refractory (R/R) cHL is salvage chemotherapy, followed by autologous stem cell transplantation (ASCT) for eligible patients [2,3,4]. The optimal salvage therapy regimen has not yet been defined, due to the lack of comparative studies [1]. A recent study by the German Hodgkin Study Group (GHSG) evaluated the outcomes of current salvage chemotherapies in R/R cHL patients, revealing a 10-year progression-free survival (PFS) rate of 48.2% and an overall survival (OS) rate of 59.4% after the first relapse, with notably inferior outcomes for those not receiving ASCT. Additionally, nearly half of patients do not achieve a complete response and approximately 50% of R/R cHL patients experience subsequent relapses [5]. A meta-analysis showed the importance of achieving a complete metabolic response (CMR) on 18FDG PET-CT (PET-CT) before proceeding to ASCT, resulting in a PFS of 55–85% [6]. The addition of 16 cycles of brentuximab vedotin (BV) maintenance post ASCT for patients at higher risk of relapse (defined as those with primary refractory disease, early relapse, or extranodal disease at relapse) has become the standard of care due to an improvement of 5 y-PFS from 41% to 59%, demonstrated in the AETHERA trial [7].

Different studies on R/R cHL have shown promising outcomes in patients who received salvage therapy with anti-PD1 antibodies compared to conventional chemotherapies [8]. In the CheckMate 744 trial, a high CMR rate of 59% was achieved after induction with nivolumab and BV before consolidation with ASCT [9]. Another trial investigated four cycles of nivolumab and BV as first salvage before ASCT, with a pre-ASCT overall response (OR) rate of 85% and a complete response rate of 67% [10].

Even though the KEYNOTE-204 study has proven the activity of pembrolizumab in the R/R cHL setting [11], to the best of our knowledge, no data are currently available on the combination of BV and pembrolizumab in R/R cHL, except for a retrospective analysis published by our group in 2022, which showed promising results, with a pre-ASCT CMR rate of 80% [12].

## 2. Materials and Methods

In this single center study, we retrospectively collected data from adult patients with R/R cHL, who started salvage treatment with BV (1.8 mg/kg IV every 3 weeks) and pembrolizumab (200 mg IV every 3 weeks) between 1 January 2019 and 31 December 2022 at the Jules Bordet Institute. We excluded patients whose initial treatment plan did not include intensification with high-dose chemotherapy (HDCT) and ASCT followed by BV maintenance for a total of 16 cycles, including the pre-ASCT cycles. This study was conducted according to the Declaration of Helsinki and approved by the local ethics committee. 

The primary outcomes were the OR rate and CMR rate, as defined by the Lugano 2014 criteria [13], before proceeding to ASCT. Secondary outcomes included response rates at the end of treatment, progression-free and overall survival estimated by Kaplan–Meier curves, toxicities, and feasibility of stem cell collection after induction therapy. Toxicities were reported according to the Common Terminology Criteria for Adverse Events (CTCAE) score v.5.0 [14].

## 3. Results

### 3.1. Patient Characteristics

We identified 24 adult patients with R/R cHL who received salvage treatment with BV and pembrolizumab with the subsequent intent of intensification with HDCT consisting of BEAM (carmustine, etoposide, cytarabine, and melphalan), followed by ASCT and BV maintenance. R/R cHL was proven by biopsy in all patients and the extent of the disease was mapped by PET-CT before the start of treatment. 

The median age was 30.2 (20.5–52.6) years. Our patient population presented a male predominance (79.2%). The median time since initial diagnosis was 2.6 (0.8–16.2) years, in which patients received a median of 3 (2–5) prior treatments. The types of prior treatment are listed in Table 1. Primary refractory or early relapsed (<12 months) diseases were present in 70% of patients, while 87.5% presented advanced stage (III-IV) disease at referral. Median follow-up time was 13.3 (6.37–39.9) months. Table 1 provides a detailed summary of patient characteristics.

### 3.2. Treatment and Outcomes

**Figure 1:**
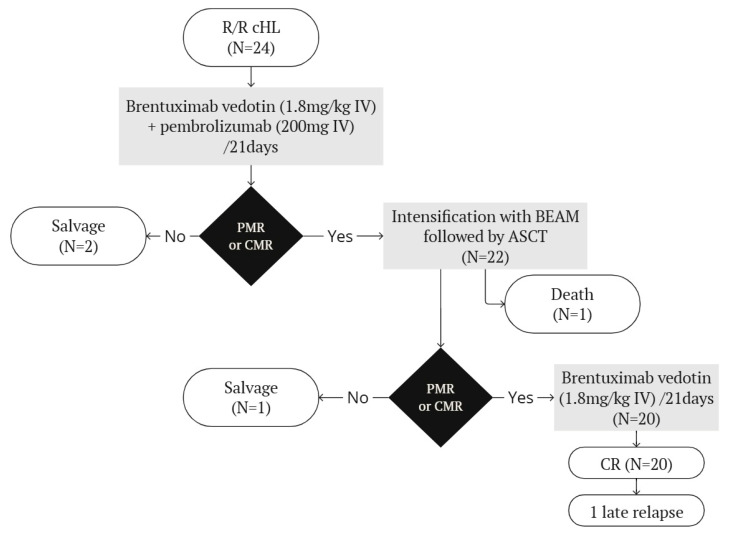
Treatment plan and outcomes. ASCT = autologous stem cell transplantation. BEAM = carmustine, etoposide, cytarabine, melphalan. CMR = complete metabolic response. PMR = partial metabolic response.

An overview of the treatment plan and outcomes are shown in Figure 1.

Though treatment plans originally included only two cycles of BV and pembrolizumab, due to the COVID-19 pandemic, intensification with HDCT and ASCT were deferred in many patients, resulting in a median of three induction cycles of BV and pembrolizumab (range 2–6).

21 patients were evaluated by PET-CT after 2 cycles, of which 95.2% achieved an overall response and 81.0% a CMR. All patients had a PET-CT evaluation before ASCT, which showed an OR rate of 91.7% and a CMR rate of 87.5%. The 22 patients achieving at least a partial metabolic response (PMR) all proceeded to HDCT followed by ASCT. Two patients with progressive disease received further salvage treatments. One patient presenting a Deauville score of 5 and stable disease after two cycles was subsequently treated with nivolumab and gemcitabine before receiving a tandem auto and allogeneic stem cell transplantation. He remained in stable disease and died twelve days after infusion of allogeneic stem cells of infectious complications and veno-occlusive hepatic disease. One patient lost a CR after receiving a prolonged induction due to the COVID-19 pandemic, and was treated with localized radiotherapy and a tandem auto- and allotransplant. Interpretation of end-of-treatment PET-CT was complicated due to a miliary tuberculosis, but at last follow-up, the patient was considered to be in complete remission.

Stem cell collection was performed at a steady state and was successful in all patients, with a mean number of collected CD34+ cells of 4.40 × 10^6^/kg body weight (ranging from 2.43 to 7.29 × 10^6^ cells/kg). Five patients (20.8%) needed two apheretic procedures to reach the target. The mobilizing agent plerixafor was added in 17 patients (70.8%) in order to obtain an adequate amount of CD34+ cells.

One patient died of septic shock during prolonged aplasia post HDCT, due to hemophagocytic lymphohistiocytosis (HLH). 

Following hematological recovery from ASCT, 21 patients started maintenance therapy with BV for a planned total of 16 administrations including pre-ASCT cycles. Evaluation by PET-CT was performed around 90 days after ASCT and at the end of maintenance treatment. One patient presented progressive disease at the first post-ASCT PET-CT evaluation, and subsequently received another salvage treatment with gemcitabine and nivolumab before proceeding to allogeneic stem cell transplantation. The patient died 105 days after allotransplantation due to multiple transplant-related complications. At the end of maintenance, the 20 remaining patients were all in complete remission. Eighteen patients (90%) had a DS1 on end-of-treatment PET-CT. One patient presented a DS4 due to persistent metabolic activity in a pulmonary lesion, which progressively diminished at each subsequent control, but did not resolve. Finally, persistent lymphoid disease was excluded by pulmonary biopsy. Another patient had a DS5 at the end of treatment, due to the apparition of pelvic adenopathies, which had spontaneously disappeared on subsequent PET-CT control and were thus considered of inflammatory origin. We recorded one late relapse at 28 months after the end of treatment, for which a salvage therapy with BV and pembrolizumab was successful in achieving partial remission. The intention is to proceed with radiotherapy on the persisting lesions, followed by an allogeneic stem cell transplantation.

Overall, we documented one treatment-related death and four treatment failures. The Kaplan–Meier analysis revealed an estimated OS rate of 91.7% (95% CI 70.6–97.8%) and a PFS rate of 83.3% (95% CI 61.5–93.4%) at one year.

### 3.3. Toxicities

The types and grades of the recorded toxicities are listed in Table 2.

During salvage therapy with BV and pembrolizumab, eight grade 4 toxicities were recorded in four patients. Notably, most grade 3 and 4 toxicities were hematological. Grade 3–4 anemia and neutropenia were managed with EPO and G-CSF, respectively. One patient presented a thyrotoxicosis secondary to a thyroiditis after the third cycle, which resolved spontaneously but imposed an interruption of pembrolizumab. Another patient developed a HLH thirteen days after the first administration, in the context of an asymptomatic COVID-19 infection. This episode was associated with a grade 3 anemia, a grade 4 thrombocytopenia and neutropenia, and biological and radiological pancreatitis without clinical symptoms, assumed to be secondary to hypertriglyceridemia. The patient showed a favorable response to treatment with etoposide and dexamethasone. For the remaining two cycles, BV and pembrolizumab were given in alternation without any new events. Another patient suffered grade 3 arthralgia, motivating an interruption of pembrolizumab after three cycles. 

After intensification with high-dose chemotherapy followed by ASCT, patients presented an expected high rate of hematological toxicities and febrile neutropenia. Duration of aplasia ranged from 7 to 101 days with a median of 10.5 days. One patient did not recover from aplasia due to HLH and died of septic shock. Other serious adverse events included one Cryptococcus neoformans meningitis, which fully recovered after treatment with amphotericin B and fluconazole, and one inflammatory pneumonitis, which resolved spontaneously with only supportive treatment.

During maintenance, dose reductions for toxicity were necessary in 16 patients (80%). BV maintenance was interrupted early (after 8 cycles of the 12 scheduled) in 1 of these patients, due to a persistent grade 3 peripheral neuropathy, despite dose reduction. The reasons for dose reduction are summed up in Table 3.

## 4. Discussion

The body of evidence for the use of checkpoint inhibitors in R/R cHL is primarily derived from non-controlled phase II trials, with the KEYNOTE-204 study as a notable exception [11]. The relative scarcity of R/R cHL patients and the heterogeneity of available salvage treatment approaches represent significant challenges for direct comparisons in randomized trials. Despite these limitations, the combination of checkpoint inhibitors and BV appears to be a valuable treatment option for R/R cHL. 

In this heavily pretreated population with poor prognostic factors, our salvage treatment regimen with BV and pembrolizumab has demonstrated a high efficacy. All patients in this study had previously received multiple lines of treatment and were classified as high risk per the AETHERA trial criteria, which supports the use of post-ASCT BV maintenance [7]. Our proposed salvage therapy achieved an impressive OR rate of 91.7% and a pre-ASCT CMR rate of 87.5%. Moreover, these are rapid responses, as demonstrated by the high OR rate observed after only two cycles. Even if it is impossible to perform comparison among different studies, these outcomes are higher than those reported in a multicenter phase 1–2 study for nivolumab and BV as first-line salvage therapy in a cohort with less high-risk patients, which yielded pre-ASCT OR and CMR rates of 85% and 67%, respectively [10]. Likewise, the CheckMate 744 study reported a CMR in 59% of patients after four cycles of nivolumab plus BV in a younger, less high-risk population [9]. Importantly, stem cell collection was not compromised by this salvage regimen, facilitating subsequent consolidation with ASCT, thus further improving the chances of good long-term outcomes.

The combination of pembrolizumab and BV was also well tolerated in our cohort. Out of 24 patients, only 4 experienced grade 4 toxicities during salvage, which indicates a relatively manageable safety profile. Treatment adaptations during salvage were necessary for three patients. One patient required a one-time omission of pembrolizumab due to thyrotoxicosis. Another patient received sequential administration of BV and pembrolizumab for HLH. A third patient had a one-time omission of pembrolizumab due to grade 3 arthralgia.

However, there were notable adverse events, including two incidences of HLH occurring during or shortly after the salvage treatment with BV and pembrolizumab. One patient succumbed to HLH, a severe condition that, while potentially related to treatment-induced dysimmunity, is also associated with lymphoma itself [15]. Notably, this complication has already been described after the combination of nivolumab and BV in the setting of R/R cHL [16]. Additionally, the second patient who experienced HLH during salvage also presented with inflammatory pneumonitis post ASCT, and immune-mediated thrombocytopenia during maintenance therapy, suggesting a broader inflammatory dysregulation, possibly linked to the treatment regimen. Perhaps, specific patient- or disease-related factors might predispose patients to such an immune dysregulation, but these remain yet to be identified. 

Our study has several limitations that warrant consideration. Firstly, as a retrospective analysis, the study relies on data from referring institutions and physicians’ notes, which sometimes included missing, incomplete, or imprecise information. In addition, the grading of severity for certain toxicities, such as neuropathy and myalgia, is dependent on subjective descriptions in the treating physicians’ notes. Additionally, the analyzed cohort was small, limiting the ability to draw definitive conclusions. The COVID-19 pandemic further complicated the study, leading to deviations from the planned treatment regimens, which could have impacted patient outcomes. Finally, the follow-up period was relatively short, as patients were typically referred back to their primary institutions upon completion of maintenance therapy, limiting the ability to assess long-term outcomes and late-onset toxicities.

Considering their promising efficacy, these novel agents might be integrated into first-line treatment in the future. This may lead to a reduction in the incidence of R/R cHL. However, their pre-existing exposure to checkpoint inhibitors and BV could ultimately limit their utility in the R/R cHL setting. 

## 5. Conclusions

For heavily pretreated R/R cHL patients, our data suggest that salvage therapy with BV and pembrolizumab before ASCT followed by BV maintenance is a highly active strategy, with manageable toxicities. The promising results of this novel treatment strategy should encourage more robust and controlled studies with greater sample size and longer follow-up to better define safety and efficacy profiles. 

## Figures and Tables

**Table 1:** Baseline characteristics of patients (N = 24).

Characteristics	
Sex, n (%)	
Male	19 (79.2%)
Female	5 (20.8%)
Median age at initial diagnosis, years (range)	28.2 (7.6–49.3)
Median age at referral, years (range)	30.2 (20.5–52.6)
Median time from diagnosis to referral, years (range)	2.6 (0.8–16.2)
Ann Arbor stage at initial diagnosis, n (%)	
II	5 (20.8%)
III	4 (16.7%)
IV	15 (62.5%)
Number of prior treatments, n (%)	
2	4 (16.7%)
3	14 (58.3%)
4	5 (20.8%)
5	1 (4.2%)
Types of prior treatments as first line, n (%)	
ABVD	9 (37.5%)
ABVD → BEACOPP-like	2 (8.3%)
BEACOPP-like	7 (29.2%)
BEACOPP-like → ABVD	3 (12.5%)
CHOP	3 (12.5%)
In subsequent lines, n (%)	
ABVD	2 (8.3%)
BEACOPP-like	3 (12.5%)
DHAC-like	20 (83.3%)
BeGeV-like	15 (62.5%)
ICE	3 (12.5%)
rituximab	1 (4.2%)
radiotherapy	5 (20.8%)
PD1-inhibitor	0 (0.0%)
brentuximab vedotin	2 (8.3%)
ASCT	1 (4.2%)
Status after front-line therapy, n (%)	
Primary refractory	14 (58.3%)
Early relapse (<12 months)	3 (12.5%)
Late relapse (≥12 months)	7 (29.2%)
Disease burden at referral, n (%)	
Stage III–IV	21 (87.5%)
Extra nodal disease	20 (83.3%)
None of the above	3 (12.5%)
Median follow-up time, months (range)	13.3 (6.4–39.9)

ABVD: Adriamycin, Bleomycin, Vinblastine, Dacarbazine; BEACOPP-like: BEACOPP (Bleomycin, Etoposide, Adriamycin, Cyclophosphamide, Vincristine, Procarbazine, Prednisone) standard or escalated, ACVBP (Adriamycin, Cyclophosphamide, Vindesine, Bleomycin, Cytarabine), MDH2003 (Etoposide, Bleomycin, Vinblastine); BeGeV-like: BeGeV (Bendamustine, Gemcitabine, Vinorelbine), benda-GVD (Bendamustine, Gemcitabine, Vinorelbine, Doxorubicin), IGeV (Ifosfamide, Gemcitabine, Vinorelbine), GVD (Gemcitabine, Vinorelbine, Doxorubicin); CHOP: Cyclophosphamide, Doxorubicin, Vincristine, Prednisone; DHAC-like: DHAC (Dexamethasone, Cytarabine, Carboplatin), DHAP (Dexamethasone, Cytarabine, Cisplatin), DHAOx (Dexamethasone, Cytarabine, Oxaliplatin); ICE: Ifosfamide, Carboplatin, Etoposide.

**Table 2:** Types and grades of toxicities.

	Grade 1	Grade 2	Grade 3	Grade 4	Grade 5
**Salvage with BV and pembrolizumab (N = 24)**					
Total	21	14	11	8	0
Hematological					
Neutropenia	0	1	1	3	0
Anemia	7	5	4	0	0
Thrombocytopenia	4	5	0	1	0
Lymphopenia	7	2	5	2	0
Thyroid					
Hypothyroidism	2	0	0	0	0
Hyperthyroidism	0	0	0	1	0
Myalgia/arthralgia	1	0	1	0	0
Pancreatitis	0	1	0	0	0
Hemophagocytic lymphohistiocytosis	0	0	0	1	0
**Intensification with BEAM and ASCT (N = 22)**					
Total	4	12	29	67	1
Neurological					
Autonomous	0	1	0	0	0
Hematological					
Neutropenia	0	0	2	20	0
Anemia	2	9	9	2	0
Thrombopenia	1	0	0	21	0
Lymphopenia	0	1	0	21	0
Infectious	0	0	18	1	1
Thyroid					
Hypothyroidism	1	1	0	0	0
Pneumonitis	0	0	0	1	0
Hemophagocytic lymphohistiocytosis	0	0	0	1	0
**BV maintenance (N = 20)**					
Total	34	17	5	1	0
Neurological					
Autonomous	1	3	0	0	0
Peripheral	6	3	1	0	0
Hematological					
Neutropenia	6	3	2	1	0
Anemia	3	5	0	0	0
Thrombopenia	7	1	1	0	0
Lymphopenia	9	0	1	0	0
Thyroid					
Hypothyroidism	0	1	0	0	0
Myalgia/arthralgia	2	1	0	0	0

**Table 3:** Reasons for dose reduction during maintenance (N = 20).

Reason	Number of Patients, n (%)
Peripheral neuropathy	8 (40%)
Arthralgia/myalgia	3 (15%)
Autonomous neuropathy	3 (15%)
Grade 3 thrombopenia	1 (5%)
Grade 4 neutropenia	1 (5%)

## Data Availability

The data that support the findings of this study are available from the corresponding author upon reasonable request. Due to privacy and ethical restrictions, certain data may be subject to confidentiality agreements.

## Abbreviations

The following abbreviations are used in this manuscript:

ASCT	Autologous stem cell transplantation
BV	Brentuximab Vedotin
cHL	Classical Hodgkin lymphoma
CMR	Complete metabolic response
CTCAE	Common Terminology Criteria for Adverse Events
DS	Deauville score
HDCT	High-dose chemotherapy
HLH	Hemophagocytic lymphohistiocytosis
OR	Overall response
OS	Overall survival
PET-CT	18FDG PET-CT
PFS	Progression-free survival
R/R	Relapsed or refractory
